# iTIME.219: An Immortalized KSHV Infected Endothelial Cell Line Inducible by a KSHV-Specific Stimulus to Transition From Latency to Lytic Replication and Infectious Virus Release

**DOI:** 10.3389/fcimb.2021.654396

**Published:** 2021-04-14

**Authors:** Stephen J. Dollery, Tania D. Maldonado, Eric A. Brenner, Edward A. Berger

**Affiliations:** Laboratory of Viral Diseases, National Institute of Allergy and Infectious Diseases, National Institutes of Health, Bethesda, MD, United States

**Keywords:** Kaposi's sarcoma-associated herpesvirus/ HHV-8/Kaposi's sarcoma, viral tropism, time, endothelial cell line, inducible, latency, lytic replication

## Abstract

Kaposi’s sarcoma-associated herpesvirus (KSHV/HHV-8) is the causative agent of Kaposi’s sarcoma and two B cell lymphoproliferative disorders: primary effusion lymphoma and KSHV-associated multicentric Castleman’s disease. These distinct pathologies involve different infected cell types. In Kaposi’s sarcoma, the virus is harbored in spindle-like tumor cells of endothelial origin, in contrast with the two pathologies of B cells. These distinctions highlight the importance of elucidating potential differences in the mechanisms of infection for these alternate target cell types and in the properties of virus generated from each. To date there is no available chronically KSHV-infected cell line of endothelial phenotype that can be activated by the viral lytic switch protein to transition from latency to lytic replication and production of infectious virus. To advance these efforts, we engineered a novel KSHV chronically infected derivative of TIME (telomerase immortalized endothelial) cells harboring a previously reported recombinant virus (rKSHV.219) and the viral replication and transcription activator (RTA) gene under the control of a doxycycline-inducible system. The resulting cells (designated iTIME.219) maintained latent virus as indicated by expression of constitutively expressed (eGFP) but not a lytic phase (RFP) reporter gene and can be sustained under long term selection. When exposed to either sodium butyrate or doxycycline, the cells were activated to lytic replication as evidenced by the expression of RFP and KSHV lytic genes and release of large quantities of infectious virus. The identity of the iTIME.219 cells was confirmed both phenotypically (specific antigen expression) and genetically (short tandem repeat analysis), and cell stability was maintained following repeated serial passage. These results suggest the potential utility of the iTime.219 cells in future studies of the KSHV replication in endothelial cells, properties of virus generated from this biologically relevant cell type and mechanisms underlying KSHV tropism and pathogenesis.

## Introduction

Since its initial discovery (Chang and Moore, 2014), Kaposi’s sarcoma-associated herpesvirus (KSHV, human herpesvirus 8) has been implicated in distinct malignancies and lymphoproliferative disorders commonly associated with HIV/AIDS (Goncalves et al., 2017; Hussein et al., 2018; Weed and Damania, 2019). The various pathologies of this gammaherpesvirus are associated with different infected cell types. In Kaposi’s sarcoma (KS) (Cesarman et al., 2019), the most common tumor in AIDS patients, the virus is harbored latently in spindle-like tumor cells found within KS lesions; these spindle cells are of endothelial origin. By contrast, the two KSHV-associated lymphoproliferative disorders, primary effusion lymphoma (PEL) (Shimada et al., 2018) and the plasmablastic form of multicentric Castleman’s disease (MCD) (Koga et al., 2017), involve KSHV-positive B lymphocytes. Moreover, B cells constitute major reservoirs of latent KSHV persisting in infected individuals and likely represent the source of virus oropharyngeal shedding and transmission by saliva (Minhas and Wood, 2014).

Diverse *in vitro* models involving the relevant target cell lineages have been developed in efforts to unravel the mechanistic complexities of KSHV infection and tropism (McAllister and Moses, 2007; Kang and Myoung, 2017; Dollery, 2019). PEL-derived B cell lines have played important roles in KSHV research, including as a source of infectious virus by induction with pleiotropic agents. Whereas extensive KSHV infection studies have been performed with primary human B cells from various tissue sources, transformed B cell lines have proven largely resistant to infection with cell-free virus; some recent exceptions have been reported for specific B cell lines, with the establishment of cells chronically infected with recombinant reporter viruses that can be induced by biologically relevant stimuli to produced infectious virus (Kati et al., 2013; Dollery et al., 2014). Regarding the other major KSHV target cell type, efficient infection with a reporter virus has been reported for primary endothelial cells (Jeffery et al., 2013) as well as an immortalized derivative (Lagunoff et al., 2002). A stable KSHV-infected endothelial cell line capable of robust infectious virus production upon activation of a KSHV-specific trigger would be particularly valuable.

Our starting point for this effort was the stable human endothelial cell line TIME (telomerase-immortalized microvascular endothelial), which is capable of continuous proliferation while retaining the characteristic surface markers of endothelial cells (Venetsanakos et al., 2002). TIME cells have proven to be a valuable *in vitro* model in KSHV research (McAllister and Moses, 2007). Particularly relevant for the present study are the early demonstrations (Lagunoff et al., 2002; Bechtel et al., 2003) that TIME cells are highly susceptible to infection by cell-free KSHV virions; the infected cells can be induced to transition from latent to lytic phase and production of infectious virus by treatment with phorbol ester or by ectopic expression of the KSHV replication and transcriptional activator (K-RTA), the lytic switch protein product of the viral ORF50 gene. We describe herein the development of a novel cell line designated iTIME.219, using an approach similar to that previously reported for a different parental cell line (Myoung and Ganem, 2011). The features of iTIME.219 cells suggest its potential value in studies of KSHV tropism and pathogenesis.

## Materials and Methods

### Cells and Reagents

Endothelial (TIME, TIME.219, iTIME.219) cells were cultured in VascuLife^®^ Basal Medium supplemented with the LifeFactors^®^ kit and 12.5 µg/ml of Blasticidin S HCL (Life Technologies). Epithelial (Vero, iSLK.219) cells were grown in DMEM media supplemented with 10% FBS (Sigma) and 5% L-Glutamine and 5% Penicillin Streptomycin (Quality Biological). Maintenance of KSHV infected cell lines (TIME.219, iTIME.219, iSLK.219) involved the addition of 10 µg/ml puromycin (Mirus Bio). A 200 µg/ml Geneticin (G418) (Life Technologies) selection was included for iTIME.219 and iSLK.219.

### Development of the Endothelial Producer Cells

KSHV infections were performed with recombinant strain KSHV.219 (rKSHV.219) (Vieira and O’Hearn, 2004). The virus was generated from iSLK.219 cells that were propagated in the presence of puromycin and induced to produce cell-free KSHV as described previously by others (Myoung and Ganem, 2011) Virus infectious units were established by titration on 293F cells (Dollery et al., 2014).

iTIME cells were generated by infecting subconfluent TIME cells with rKSHV.219 at an MOI 3 and allowing infection to proceed for three days. Following initial infection, cells were selected by culture in media containing 10 µg/ml of puromycin. For transductions, pRetroX-Tet-On Advanced (rtTA plasmid from Clonetech) and pRetroX-Tight-Hyg-RTA (RTA gene cloned into pRetroX-Tight-Hyg from Clonetech) were separately transfected into GP2-293 cells along with an RD114 envelope vector. Culture supernatants containing retroviral particles were harvested after 2 days. TIME.219 were first transduced with retrovirus encoding rtTA in the presence of polybrene (8 µg/ml) for 3 h at 1,500 RPM (Thermo Scientific Heraeus Multifuge X1R with TX-400 rotor). Cells harboring the rtTA construct were selected by G418 at 200 µg/ml. The cells were then transduced again with retrovirus containing the pRetroX-Tight-Hyg-RTA construct. Transductants were selected by hygromycin at 400 µg/ml. Cells were maintained in media with 200 µg/ml G418 and 10 µg/ml puromycin.

### Immunocytochemistry of Cell Type-Specific Markers

The following monoclonal antibodies (mAbs) or isotype control were employed for cell marker detection: for endothelial cells, the anti-CD31 rabbit monoclonal antibody (mAb) EPR17259 (Abcam, 1:50 dilution); for epithelial marker detection, the anti-pan cytokeratin mAb AE1/AE3 (Abcam, 1:10 dilution). Cells were fixed with ice cold methanol (CD31) or 3% paraformaldehyde (cytokeratin). To permeabilize samples for cytokeratin detection, 0.2% Saponin (CalbioChem) in 1% BSA (Sigma)/PBS was employed. Samples were blocked with 2% goat serum plus 1% BSA in PBS and then incubated for 1 h with primary antibodies. Cells were then washed with 1% BSA in PBS and blocked with previously described serum. For detection, cells were probed with either Alexaflour (AF) 647 goat anti-rabbit IgG (H + L) (Invitrogen) or Alexaflour (AF) 647 goat anti-mouse IgG (H + L) (Invitrogen). After two washes, nuclei were stained with DAPI (1/1,000) (AAT Bioquest) for 5 min. All micrographs were taken with the EVOS FL Auto 2 (Invitrogen) at 10× using the 2.0 Imaging System. Images were enhanced with Adobe Photoshop CC 2015 software.

### Antibody Staining of Viral Proteins

For chronically infected and newly infected cells, iTIME.219, TIME, and infected Vero cells were plated overnight at 8.3 × 10^4^ cells per well in a 24 well plate. Cells were washed with 1% BSA in PBS. Samples were then fixed with 3% PFA for 10 min and permeabilized with 0.2% Triton X-100 for 25 min. Samples were washed twice and blocked with 5% BSA/PBS for 30 min. Anti-LANA mAb LN53 (EMD Millipore) (1:100 dilution) was added to cells and incubated for 1 h. Cells were then washed in 1% BSA in PBS and blocked with 2% goat serum in 1% BSA/PBS for 30 min. Samples were then incubated with AF 647 Goat anti-Rat (Invitrogen) at a 1:500 dilution. Representative micrographs were taken with an EVOS FL Auto 2 at 20× using the 2.0 Imaging System. For glycoprotein detection by fluorescence microscopy, uninduced and induced iTIME.219 cells were plated at a concentration of 1 × 10^5^ cells per well, washed with 1% BSA/PBS, and fixed with 3% paraformaldehyde. Samples were blocked with 5% BSA/PBS and incubated with a 1:100 dilution of anti-gH mAb YC15 (Cai and Berger, 2011) or ant-K8.1 mAb 4C3 (Zhu et al., 1999) for 45 min. Cells were again washed with 1% BSA/PBS and blocked with 2% goat serum prepared in 1% BSA/PBS. For detection, samples were incubated for 20 min with Alexaflour (AF) 647 Goat anti-Mouse IgG (H + L) (Invitrogen, 1:2,000). All infected, newly infected, uninduced, and induced samples were stained with DAPI as described before. Micrographs were taken with an EVOS FL Auto 2 and processed using the EVOS 2.0 Imaging System and the Adobe Photoshop CC 2015. For viral glycoprotein detection by flow cytometry, cells were dislodged from monolayers with *CellStripper* Dissociation Reagent (Thermo), fixed with 3% PFA, and 1 × 10^6^ cells per sample were then stained and as above with the addition of centrifugation to pellet the samples before solution changes. Analysis was performed using a FACSCanto II (BD Biosciences), and data analysis was performed using FlowJo (Treestar). For the detection of eGFP or DS-RED detection alone, induced and/or infected cells were prepared for flow cytometry as above, with antibody probing steps and blocking steps omitted. Analysis was again performed using a FACSCanto II (BD Biosciences), and data analysis was performed using FlowJo (Treestar).

### Induction and Infection of Cell Lines

To test induction of the KSHV lytic cycle and virion production of latently infected cells, engineered cell lines and controls were exposed to doxycycline (DOX) and/or sodium butyrate (SB) in media without selective agents puromycin or G418. The indicated concentrations of DOX (µg/ml) and SB (mM) were added to the cells plated overnight in six well plates (3.5 × 10^5^ cells in 2 ml per well). Cells were incubated with reagents for 3 days. Following induction, micrographs of induced cells were captured using an EVOS FL Auto 2 (Texas Red cube) (10×) and processed (EVOS 2.0 Imaging System and the Adobe Photoshop CC 2015). Immediately following microscopy, supernatants were harvested to quantitate progeny virions. 2 ml of the supernatant was collected and clarified by pelleting debris at 3,000 rpm for 10 min at 4°C (Thermo Scientific Heraeus Multifuge X1R with TX-400 rotor). Progeny virions in the supernatant were then concentrated by pelleting at 16,000 ×g at 4°C for 3.5 h. Concentrated virus was then resuspended in 200 ul of Vero media, serially diluted in Vero media and titered on Vero cells. Micrographs of infected Vero cells and controls were taken at 3 days post-exposure to supernatant with an EVOS FL Auto 2 (GFP cube) (10×) and processed (EVOS 2.0 Imaging System and Adobe Photoshop CC 2015). Cells were analyzed by flow cytometry using a BD FACSCanto II cytometer, and the data were processed using DIVA and FloJo version 10.4.2. The data sets presented for all infection and induction experiments are representative of at least three independent repeat experiments.

### Statistical Analysis

Statistical significance was calculated using a two-tailed unpaired student's t-test.

### Short Tandem Repeat Analysis

For STR analysis and identification of the TIME, TIME.219, and iTIME.219 cells, genomic DNA was isolated using the DNeasy Blood & Tissue Kit ^®^ (Qiagen). Isolated DNA was sent on ice to the Genetic Resources Core Facility (GRCF) at the Johns Hopkins University School of Medicine for STR analysis using the Promega PowerPlex 16 HS system.

## Results

### Generation of Chronically Infected Endothelial Cells Based on the TIME Cell Line

In order to generate a stable KSHV-infected endothelial cell line capable of robust production of infectious virus upon KSHV-specific induction, we subjected TIME cells to a modification of the approach reported for production of the iSLK.219 cell line (Myoung and Ganem, 2011) (see *Materials and Methods*). TIME cells were first infected with the recombinant virus rKSHV.219 (Vieira and O’Hearn, 2004), which contains two fluorescent reporter genes: the eGFP gene linked to the constitutive elongation factor 1*α* cellular promoter plus the RFP gene under control of the KSHV early lytic-phase PAN promoter. The recombinant virus also contains the puromycin-resistance gene linked to the constitutive Rouse sarcoma virus promoter, which enables positive selection of cells harboring the KSHV episome. The KSHV-infected TIME cells were subjected to puromycin selection to generate a cell population designated TIME.219; these cells retain the recombinant virus genome indefinitely when maintained under selection (tested up to passage 30, data not shown). In order to confer on TIME.219 cells a KSHV-specific system for activating the virus from latent to lytic phase, we introduced genetic components whereby expression of KSHV RTA, the lytic transactivator protein, is induced upon addition of the tetracycline analog doxycycline (Dox). These cells, designated iTIME.219, were expanded and maintained under continuous selection with puromycin plus G418 (see *Materials and Methods*).

**Figure 1:**
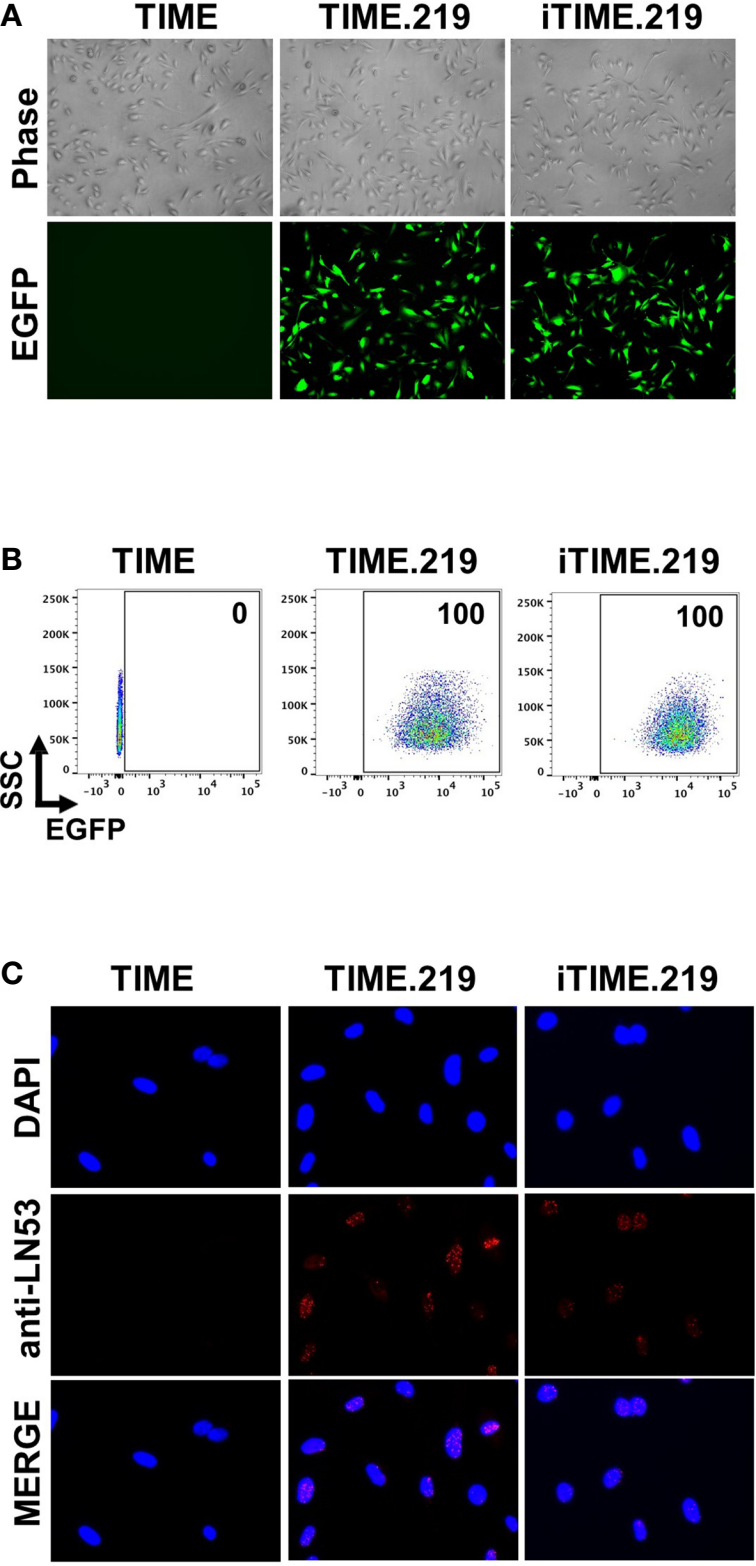
Analysis of uninduced TIME cells and the KSHV chronically infected derivatives. TIME, TIME.219, and iTIME.219 cells were analyzed for KSHV infection as assessed by expression of eGFP, the latent phase (constitutively expressed) reporter. **(A)** Phase contrast (top) and fluorescence microscopy for eGFP expression (bottom). Magnification 10×. **(B)** Infection by rKSHV.219 analyzed by flow cytometry for eGFP expression. Results are depicted as scatter graphs plotting side scatter (SSC) against log fluorescence intensity. Numbers in the upper right corners indicate the percentage of cells within the eGFP^+^ gate. **(C)** Cells were fixed, permeabilized and probed with anti-LANA mAb LN53; detection was with an Alexa-647 conjugated anti-rat antibody (orange). Nuclei were stained with DAPI (blue). Images (magnification 20×) are shown for DAPI (top), LANA (middle), and merged (bottom).

Assessment of eGFP expression in uninduced cells by either fluorescence microscopy (**Figure 1A**) or flow cytometry (**Figure 1B**) revealed that for both the TIME.219 and iTIME.219 populations, the vast majority of cells were infected with the KSHV reporter virus. To confirm true KSHV infection and its maintenance during continued selection, staining for the latency-associated nuclear antigen (LANA) was performed; punctate nuclear LANA staining is commonly used to verify the presence of KSHV episomes during the latent stage of infection. As shown in **Figure 1C** for both TIME.219 and iTIME.219, most cells displayed LANA punctate staining in regions that were also positive for the nuclear DAPI stain; by contrast, no LANA staining was observed in the nuclei of the uninfected TIME cells. These results confirm the maintenance of KSHV infection during continuous propagation and selection of the TIME.219 and iTIME.219 cells.

### Induction of KSHV Lytic Phase

**Figure 2:**
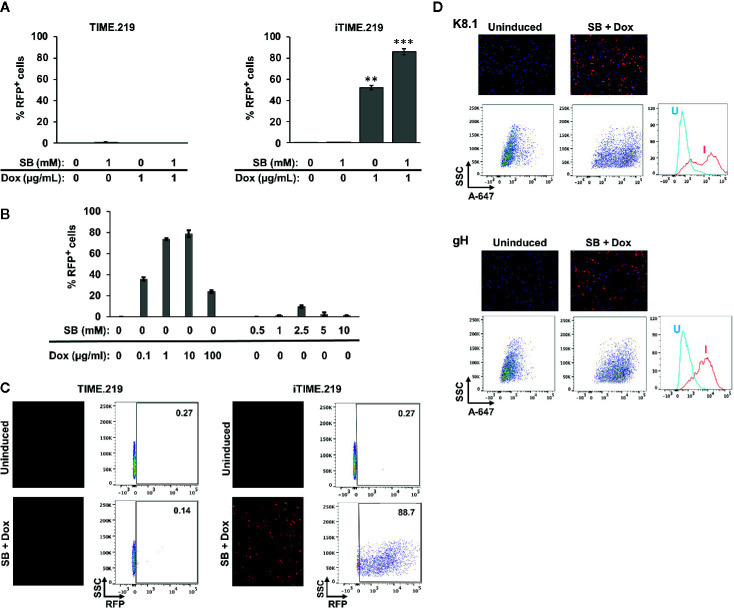
Induction of TIME.219 and iTIME.219 cells by various combinations of sodium butyrate and doxycycline. In panels **(A–C)**, induction was assessed based on expression of RFP, the lytic phase reporter. Cells were exposed for three days to the indicated concentrations of SB, DOX, or SB plus Dox. TIME.219 (left) and iTIME.219 cells (right). **(A)** Cells were analyzed for RFP expression by flow cytometry. Data are plotted as percent induced cells (% RFP^+^). **P ≤ 0.01; ***P ≤ 0.001, relative to no treatment. Error bars indicate standard error of the mean (SEM). **(B)** iTIME.219 cells were exposed to the indicated concentrations of DOX or SB for three days. Cells were analyzed for RFP expression by flow cytometry. Data are plotted as percent induced cells (% RFP^+^). **(C)** Data for a subset of cells from the experiment in panel **(A)** including TIME.219 (left) and iTIME.219 cells (right) either uninduced (upper subpanels) or induced with 1 mM SB + 1 µg/ml Dox (lower subpanels). For each cell type, the data shown are fluorescence photomicrographs (left subpanels, magnification 10×) and scatter plots (right subpanels; numbers in each upper right corner indicate percent cells within the RFP^+^ gate). **(D)** iTIME-219 induction analysis based on expression of KSHV glycoproteins K8.1 (probed with mAb 4C3, top set of panels) and gH (probed with mAb YC15, bottom set of panels), in each case for cells that were uninduced (left subpanels) and induced (1 mM SB + 1 µg/ml Dox, right subpanels). Detection was performed with an anti-mouse-AF647 conjugate. Cells were analyzed by fluorescence photomicroscopy (top subpanels) and flow cytometry (middle and bottom subpanels). For each glycoprotein, the flow cytometry plot (bottom subpanel) shows comparison of fluorescence intensities for cells that were uninduced (U) *vs*. induced (I).

We next examined these cells for their ability to transition to KSHV lytic phase, comparing uninduced and induced conditions (**Figure 2**). Two potential inducing agents were tested: sodium butyrate (SB), a non-specific compound with pleiotropic effects likely due to histone deacetylase inhibition, and Dox, a selective activator in the engineered iTIME.219 cells. We initially analyzed the expression of the lytic phase RFP reporter. Based on flow cytometry, the TIME.219 cells (**Figure 2A**, left panel) displayed no detectable induction by either agent at the doses tested, alone or in combination. The iTIME.219 cells (**Figure 2A**, right panel) also were refractory to SB alone but were efficiently induced by 1 μg/ml Dox alone (~50% RFP^+^); combination treatment resulted in even further induction (~90% RFP^+^). In more extensive dose–response analyses of iTIME.219 cells (**Figure 2B**), Dox alone at concentrations above 1 μg/ml did not further enhance RFP induction significantly; SB alone produced modest induction when increased to 2.5 or 5 mM (15–20% RFP^+^), with lesser effect at higher concentrations; induction by the combination of 1 μg/ml Dox plus 1 mM SB was not significantly improved by different concentrations of each agent (not shown). **Figure 2C** shows the corresponding data for each cell population analyzed by fluorescence microscopy (left panels) and flow cytometry plots.

We extended the RFP reporter results by testing whether KSHV late lytic phase proteins are expressed upon induction (**Figure 2D**). Immunofluorescence microscopy and flow cytometry revealed that KSHV glycoproteins K8.1 (**Figure 2D** upper panels) and gH (**Figure 2D** lower panels) were minimally detectable in uninduced iTIME.219 cells; upon induction with Dox + SB, the expression of both glycoproteins appeared to increase in the majority of cells, as evidenced by the shifted populations. These results demonstrate inducer-mediated transition of the iTIME.219 cells to the lytic phase of the KSHV infection cycle.

**Figure 3:**
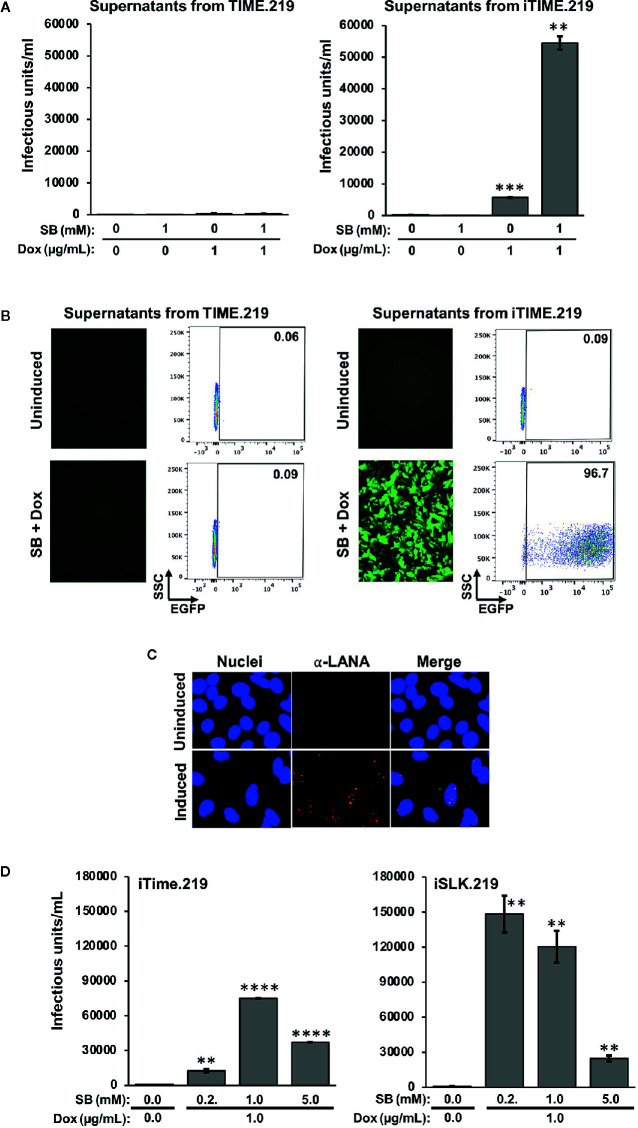
Comparison of infectious virus induction from TIME.219 and iTIME.219 cells by various combinations of sodium butyrate and doxycycline. Supernatants from the experiment in **Figure 2** were collected, clarified, concentrated, and resuspended in 200 µl of fresh media. The concentrated supernatants were analyzed for infectivity on Vero target cells as assessed by eGFP fluorescence. **(A)** Vero cells were exposed to serial dilutions of supernatants from TIME.219 cells (left panel) and iTIME.219 cells (right panel) and cultured for three days in the presence of the indicated concentrations of SB, DOX, or SB plus Dox. Infected cells were assessed by flow cytometry. Infectious unit was defined as the amount of virus needed to result in a single cell expressing eGFP. Infectious units per ml were quantitated from dilutions within the linear range of detection and are plotted on the y-axis. **(B)** Selected Vero target cells generated as described in panel **(A)**, infection based on eGFP expression was assessed by fluorescence photomicroscopy (left subpanels, magnification 10×) and flow cytometry (right subpanels; numbers in each upper right corner indicate percent cells within the eGFP^+^ gate). **(C)** LANA staining: Vero cells exposed to supernatants from uninduced (upper panels) or induced (lower panels) iTIME.219 cells were fixed, permeabilized and stained with anti-LANA mAb LN53 followed by an Alexa-647 conjugated anti-rat antibody. Nuclei were stained with DAPI (blue, left panels) LANA (orange, center panels); merged (right panels) Images shown are at 20× magnification and are cropped to further aid visualization of puncta. **(D)** Comparison of virus production from iTIME.219 and iSLK.219 cells. iTIME.219, and iSLK.219 cells were exposed to the indicated concentrations of SB and Dox, and concentrated supernatants containing virus were prepared. Vero target cells were exposed to the preparations, and infectious units were calculated as described for **(A, B)** **P ≤ 0.01; ***P ≤ 0.001; ****P ≤ 0.0001. Error bars indicate SEM.

We next assayed infectious virus production from the TIME.219 and iTIME.219 cells treated with the inducing agents employed above (**Figure 3**). Culture supernatants were collected at day 3 post-treatment, virus was concentrated by centrifugation, and the resuspended virus pellets were plated onto Vero cells, a commonly used target for KSHV infection studies. eGFP expression was used as an initial marker of Vero infection. **Figure 3A** shows that TIME.219 cells failed to generate appreciable detectable infectious KSHV upon treatment with SB or DOX, alone or in combination. By contrast, iTIME.219 cells were induced to produce infectious virus by Dox alone; SB alone was ineffective but enhanced the Dox-mediated induction. **Figure 3B** shows the corresponding fluorescence microscopy and flow cytometry analyses for the cells treated with SB plus Dox. Overall, the response patterns for induction of infectious KSHV production (**Figure 3A**) paralleled those described above for RFP expression in the induced cells (**Figures 2A, B** **)**. Further analyses of dose–response effects on infectious virus production (data not shown) yielded results similar to those noted above for RFP expression. Thus, increasing Dox alone above 1 μg/ml gave minimal improvement of yield; increasing SB alone to 2.5 or 5 mM generated low but detectable amounts of infectious virus, and virus yield from combination treatment with 1 μg/ml Dox plus 1 mM SB was not significantly improved by modifying the doses of either agent.

To verify that the supernatant-exposed Vero cells had truly entered latent phase of infection, LANA staining was performed as described above for the iTIME.219 cells. As shown in **Figure 3C**, LANA puncta were visible in the nuclei of Vero cells exposed to the supernatants from induced iTIME.219 cells, but not the uninduced cells. Taken together, the results in this section demonstrate that iTIME.219 cells are latently infected with recombinant rKSHV.219 and can be triggered to undergo the essential features of the lytic phase by activating a KSHV-specific induction pathway.

To gain perspective on the efficiency of infectious virus production, we compared the iTIME.219 cells with the previously reported iSLK.219 cells (Myoung and Ganem, 2011), which have proven valuable as a reliable source for generating the recombinant rKSHV.219 reporter virus. **Figure 3D** shows that both cell types produce robust amounts of virus, with iSLK.219 cells producing around 40% more at the previously described optimal concentrations of SB plus Dox.

### Cell Line Verification

**Figure 4:**
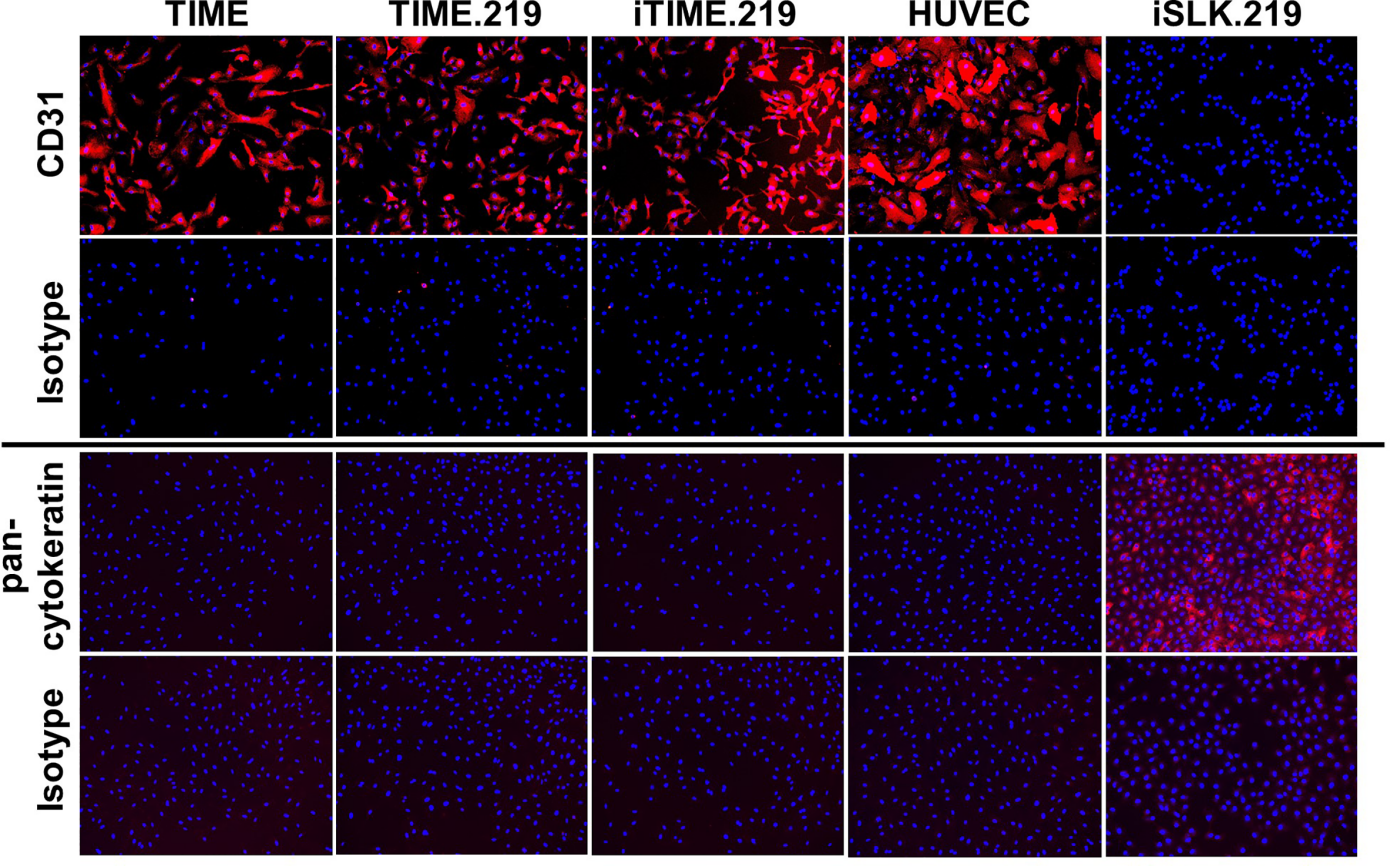
Phenotypic analysis for cell type-specific markers. The indicated cells were grown to 95% confluence, fixed, and stained with mAbs specific for CD31 (endothelial marker), or PAN cytokeratin (epithelial): or isotype-matched non-specific antibody controls. Detection was performed with an Alexa-Fluor-647-conjugated anti-mouse IgG antibody and is shown in orange. Nuclei were stained with DAPI (blue). Images are shown at 10× magnification.

In light of the concerns associated with potential cell line misidentification (Horbach and Halffman, 2017), we applied two approaches to verify the authenticity of the TIME cells and its derivatives. We first assessed immuno-phenotype, using CD31 expression as a classical endothelial cell marker and human vascular endothelial cells (HUVECs) as an endothelial cell standard; we also analyzed pan-cytokeratin as a classical epithelial cell marker, using the iSLK.219 epithelial cell line (Sturzl et al., 2013) as a standard. Corresponding isotype control antibodies were used for negative controls. As shown in **Figure 4**, the TIME, TIME.219, and iTIME.219, as well as HUVEC cells all stained positive for CD31 and negative for pan-cytokeratin, consistent with endothelial phenotype. The iSLK.219 cells showed the reciprocal immuno-staining pattern, *i.e.* negative for CD31 and positive for pan-cytokeratin, as expected for its epithelial phenotype.

We next applied the favored method for cell authentication, short tandem repeat (STR) profiling (Masters et al., 2001). This procedure was applied to genomic DNA samples prepared from TIME, TIME.219, and iTIME.219 cells and subjected to STR profiling. The profiles were compared to two databases of known STR profiles from the American Type Culture Collection (ATCC) and the Deutche Sammlung von Mikroorganismen und Zellkulturen (DSMZ). The data in **Table 1** indicate that at all nine loci, there was a 100% match between each of the TIME-derived experimental STR profiles and the TIME profiles in both databases.

**Table 1:** STR analysis of TIME cells and KSHV chronically infected derivatives.

Cell Line	Locus names								
	D5S818	D13S317	D7S820	D16S539	vWA	TH01	AMEL	TPOX	CSF1PO
TIME	11,11	9,11	8,9	9,12	16,18	6,7	X,Y	8,8	11,12
TIME.219	11,11	9,11	8,9	9,12	16,18	6,7	X,Y	8,8	11,12
iTIME.219	11,11	9,11	8,9	9,12	16,18	6,7	X,Y	8,8	11,12
TIME (DSMZ)	11,11	9,11	8,9	9,12	16,18	6,7	X,Y	8,8	11,12
TIME (ATTC)	11	9,11	8,9	9,12	16,18	6,7	X,Y	8	11,12

STR analysis was performed on the TIME, TIME.219, and iTIME.219 cells used in this study (upper three lines) and results were compared to data on TIME cells obtained from two biorepositories (lower two lines): the Deutche Sammlung von Mikroorganismen und Zellkulture (DSMZ) and the American Type Culture Collection (ATCC). The table indicates the locus names and the number of STR repeats found at each locus for the indicated cell line.

Taken together, our data verify the endothelial phenotype of the TIME-derived cells developed in this study and their genetic identity with the TIME cells reported in the established ATCC and DSMZ databases.

## Discussion

The findings presented herein demonstrate that 1) iTIME.219 endothelial cells maintain latent infection with rKSHV.219, 2) the cells can be induced to progress through the complete virus lifecycle, 3) induced cells produce robust amounts of infectious KSHV, and 4) induction can be achieved by activating the natural trigger for the latent to lytic transition, without the need for agents that promote non-specific pleiotropic effects.

To establish the iTIME.219 cells, we employed a modification of the approach previously described by Myoung at Ganem for generating a chronically infected endothelial cell line designated iSLK.219 (Myoung and Ganem, 2011), including use of the rKSHV.219 recombinant virus and incorporation of a doxycycline-inducible RTA gene. In that study, the authors employed SLK as the parental cell line based on its previous description as endothelial-derived (Herndier et al., 1994). However a subsequent study revealed SLK to be a contaminant from a renal carcinoma cell line, and the investigators noted its unsuitability for KSHV/endothelial biology and pathogenesis (Sturzl et al., 2013). Thus, the iTIME.219 cells described herein represent a novel system for investigating the various phases of the KSHV cycle in endothelial cells, for analyzing properties of virus particles generated from this critically relevant cell type, and for comparing these features in endothelial cells with those in B lymphocytes, the other major cell type involved in KSHV pathogenesis.

A particularly interesting question is the possible influence of producer cell type on KSHV tropism. There is precedent for such relationships among both animal and human gammaherpesviruses, whereby cell type-specific variations in viral glycoprotein expression result in complex effects on tropism (Gillet et al., 2015; Hutt-Fletcher, 2017). For bovine herpesvirus-4, virions produced from infected epithelial cells have high levels of the gp180 glycoprotein and are minimally infectious for CD14^+^ circulating cells, whereas virions generated from myeloid cells express reduced gp180 levels and are readily infectious for these cells; these expression differences are associated with alternative splicing of the corresponding gene (Machiels et al., 2011; Machiels et al., 2013). For the human pathogen Epstein–Barr virus, epithelial cells produce virus expressing glycoprotein complexes containing gp42, whose binding to cell surface HLA II is essential for virus fusion/entry into B lymphocytes. By contrast, B cells generate virus with relatively reduced expression of gp42 owing to its intracellular capture in the HLA II processing pathway, resulting in relative enhancement of epithelial cell infection. Thus each of these important target cell types generates virus with reciprocal relative tropism (Wang et al., 1998; Borza and Hutt-Fletcher, 2002).

Might KSHV also exploit producer cell-dependent variations in tropism in establishing infection of its major target cell populations, B lymphocytes and endothelial cells? Based on studies with a permissive B cell line and primary B cells from tonsil, we have reported (Dollery et al., 2019) that the K8.1A glycoprotein of KSHV is critical for B lymphocyte infection but dispensable for primary endothelial cells (and for other non-B cell targets, as had been well-established in the literature). This finding complemented extensive evidence in the literature demonstrating the tropism involvement of proteins encoded by positionally homologous genes in cell tropism of other gammaherpesviruses, as described above for gp180 of bovineherpesvirus-4 (Machiels et al., 2011; Machiels et al., 2013), as well as for gp350/320 of Epstein–Barr virus (Hutt-Fletcher, 2007). This raises the question of whether variations in expression or processing of K8.1 gene products in different virus producer cells play a role in KSHV tropism. The iTIME.219 endothelial cells provide a framework for investigating such questions, involving comparisons with KSHV chronically infected B cell lines that can be activated for virus production by KSHV-specific pathways (Dollery et al., 2014; Kati et al., 2015).

## Data Availability Statement

The original contributions presented in the study are included in the article/supplementary material. Further inquiries can be directed to the corresponding authors.

## Author Contributions

EBr designed and performed preliminary experiments with oversight from SD and EBe. TM continued experiments and instigated validation studies with oversight from EBe and SD. SD, TM, and EBe prepared the initial draft of the manuscript. All authors edited subsequent drafts. EBe oversaw all aspects of funding. All authors contributed to the article and approved the submitted version.

## Funding

This research was funded in part by the Division of Intramural Research of National Institute of Allergy and Infectious Diseases (ZIA AI000733-25) and by the National Institutes of Health Intramural AIDS Targeted Antiviral Program.

## Conflict of Interest

The authors declare that the research was conducted in the absence of any commercial or financial relationships that could be construed as a potential conflict of interest.
